# Characterization of gene family that mediates the adhesion of biofilms formed by *Candida tropicalis* isolated from HIV and non-HIV patients

**DOI:** 10.1186/1471-2334-12-S1-O8

**Published:** 2012-05-04

**Authors:** PM Punithavathy, Thangam Menon

**Affiliations:** 1 Dr. ALM Post Graduate Institute of Basic Medical Sciences, University of Madras, Taramani, Chennai, India

## Background

*Candida tropicalis* is an important cause of candidemia in immunocompromised patients. Biofilm formation helps the organism to establish infection. Agglutinin like sequence (ALS) genes encodes glycoproteins which are important adhesins. This study was done to detect the presence of ALS genes by PCR in *C.tropicalis* strains isolated from HIV and non-HIV patients in comparison with biofilm formation.

## Materials and methods

Yeast isolates: A total of 48 *C.tropicalis* isolates (HIV-20; non-HIV-28) were included in this study. Biofilms were formed on 96 well plates as described earlier and ALS genes were detected by PCR using specific primers.

## Results

Among the 48 *C.tropicalis* isolates, 16 out of 20 (80%) HIV isolates and 17 out of 28 (61%) non-HIV isolates were biofilm producers; 4 out of 20 HIV isolates (20%) and 11 out of 28 (39%) non HIV isolates were biofilm non-producers. Out of 48 isolates, 12/48 (25%) isolates were positive for ALS 1; 24/48 (50%) isolates were positive for ALS 2; 23/48 (48%) isolates were positive for ALS 3. Thirty four out of 48 (71%) isolates were positive for one or more ALS genes. Twenty two of the 34 (65%) were biofilm producers. Of the 14 strains which were negative for all ALS genes, 11 (79%) were biofilm producers.

## Conclusion

ALS 2 and ALS 3 genes were more common in *C.tropicalis* than ALS 1. The biofilm forming ability of the strains was independent of the presence of the ALS genes and the source of the isolates.

